# Symptoms of egg yolk‐associated food protein‐induced enterocolitis syndrome appear following prolonged cessation

**DOI:** 10.1002/jpr3.70077

**Published:** 2025-08-25

**Authors:** Yuka Okura, Masaaki Shimomura, Yutaka Takahashi, Ichiro Kobayashi

**Affiliations:** ^1^ Center for Pediatric Allergy and Rheumatology KKR Sapporo Medical Center Sapporo Japan

**Keywords:** Asymptomatic period, consumption interval, onset

## Abstract

Food protein‐induced enterocolitis syndrome (FPIES) is a nonimmunoglobulin E (IgE)‐mediated food allergy. Most patients with solid FPIES experience a period of asymptomatic intake of the causative foods before onset. This study aimed to elucidate the pattern of egg yolk (EY) ingestion that triggers FPIES. We retrospectively compared consumption intervals during the asymptomatic period to those just before the first FPIES episode in 24 patients with oral food challenge test‐confirmed EY‐FPIES, for whom complete data regarding the dates of EY consumption before onset were available. The average interval during the asymptomatic period and that between the last asymptomatic consumption and onset were 2.2 ± 2.3 (median, 1; interquartile range [IQR], 1–3; 95% confidence interval [CI], 1.75–2.68) days and 17.1 ± 12.7 (median, 13; IQR, 8–29 days; 95% CI, 11.70–22.46), respectively. The onset of FPIES is associated with prolonged cessation after asymptomatic consumption.

## Abstract (highlights)

**What is Known:**

- Food protein‐induced enterocolitis syndrome (FPIES) is a nonimmunoglobulin E (IgE)‐mediated food allergy characterized by vomiting with or without constitutional symptoms, which occur 1–4 h after intake of the causative foods.
- Most patients with solid FPIES experience a period of asymptomatic intake of the causative foods before onset.

**What is New:**

- Average interval of egg yolk (EY) consumption was 2.2 ± 2.3 days during asymptomatic periods.
- First FPIES symptoms developed after 17.1 ± 12.7 days cessation of EY.
- Onset of FPIES is associated with prolonged cessation of the causative foods in patients with EY‐associated FPIES.

## 1 INTRODUCTION

Food protein‐induced enterocolitis syndrome (FPIES) is a nonimmunoglobulin E (IgE)‐mediated food allergy that predominantly affects infants.^1^ FPIES is clinically characterized by vomiting 1–4 h after ingestion of causative foods, often accompanied by diarrhea and constitutional symptoms such as pallor, lethargy, hypotension, and hypothermia.^1^ Cow's milk is a common causative food in neonatal FPIES.^1^ Conversely, the major causes of solid FPIES are different between countries or areas; egg yolk (EY) is the most prevalent cause in Japan.^1^, ^2^ We have noticed that most patients with solid FPIES experience a period of asymptomatic intake of the causative foods before onset,^3^ indicating the role of sensitization to these foods before the development of solid FPIES. However, patterns of EY consumption before the onset of EY‐associated FPIES (EY‐FPIES) have not been analyzed in a sufficiently large sample population. In the present study, we investigated the differences in EY consumption intervals between the asymptomatic period and the period immediately preceding the first symptom of FPIES in patients with EY‐FPIES.

## 2 METHODS

This study included infants with EY‐FPIES who had complete records from their guardians detailing all EY consumption dates and who tested positive on an oral food challenge (OFC) at our hospital between April 2018 and September 2023. To evaluate potential selection bias, we analyzed the demographics of the excluded patients. The open OFC was performed by administering EY in a single portion at a median dose of 0.16 g (interquartile range [IQR], 0.11–0.20) EY protein per kg of body weight under overnight close observation at the pediatric ward of our hospital (Table 1). FPIES was diagnosed according to international guidelines.^1^ We retrospectively reviewed electronic medical records to collect data detailing EY consumption patterns, including the dates of all EY consumption from the initial introduction to the first episode, date of diagnosis, and specific IgE levels measured using the ImmunoCAP test during diagnosis. We compared the consumption intervals during the asymptomatic period to those from the last asymptomatic consumption to the first FPIES episode.

**Table 1:** Characteristics of patients with EY‐associated FPIES.

	Included cases, *n* = 24	Excluded cases, *n* = 49 (unless otherwise noted)	*p* value
Sex: male, *n* (%)	13 (54.2%)	22 (44.9%)	*p* = 0.6185
Age at first introduction of EY (days); median (IQR)		*n* = 14^a^	
	208 (188–223.5)	198.5 (188.8–218.0)	*p* = 0.6057
Age at first symptomatic episode (days); median (IQR)		*n* = 36^a^	
	236 (221.5–242.8)	236 (223.8–258.8)	*p* = 0.2711
Age at diagnosis of EY‐ FPIES (days); median (IQR)	282 (252.8–307.5)	311 (282.0–387.0)	*p* = 0.0214
Challenge doses of EY (food protein/body weight; g/kg); median (IQR)	0.16 (0.11–0.20)	0.14 (0.09–0.26)	*p* = 0.4428
EY‐specific IgE at diagnosis (U_A_/mL); median (IQR)	0.06 (0.00–0.5675)	0.00 (0.00–0.45)	*p* = 0.384
EW‐specific IgE at diagnosis (U_A_/mL); median (IQR)	0.34 (0.00−2.863)	0.26 (0.00–2.210)	*p* = 0.7172
OVM‐specific IgE at diagnosis (U_A_/mL); median (IQR)	0.00 (0.00−0.00)	0.00 (0.00–0.050)	*p* = 0.5080
Complication or history of atopic dermatitis	9 (37.5%)	27 (55.1%)	*p* = 0.2140
Complication of immediate food allergy	0	3 (6.1%)	*p* = 0.5462
Asymptomatic history of EY consumption before onset	24 (100%)		
Intervals between first introduction and first symptomatic episode (days); median (IQR)	25.5 (15.0–39.5)		
Number of asymptomatic consumptions before onset; median (IQR)	5.0 (3.25–7.0)		
Intervals during asymptomatic consumption (days); mean ± SD	2.2 ± 2.3		
Intervals between the last asymptomatic consumption and onset (days); mean ± SD	17.1 ± 12.7		

Abbreviations: EW, egg white; EY, egg yolk; FPIES, food protein‐induced enterocolitis syndrome; IQR, interquartile range; OVM, ovomucoid; SD, standard deviation.

a The number of patients whose precise data are available.

Statistical analyses were performed using GraphPad Prism 10 software (GraphPad Software). The Mann–Whitney *U* test was performed to compare the EY consumption intervals during the asymptomatic period to the time interval from the last asymptomatic consumption to the first episode. Statistical significance was set at *p* < 0.05.

### 2.1 Ethics statement

This study is approved by the institutional ethics committee of KKR Sapporo Medical Center (#2023‐04). Written informed consent was obtained from the guardians of patients before each OFC.

## 3 RESULTS

A total of 73 patients were diagnosed with EY‐FPIES. Emesis that occurred 1–4 h after the ingestion of EY was invariably the first symptom with or without accompanying diarrhea, pallor, or lethargy. Among the patients, 49 were excluded due to incomplete information regarding the date of EY consumption. Finally, 24 patients met the inclusion criteria and were enrolled. The baseline characteristics of the included and excluded patients are presented in Table 1. The median ages of the included patients at the first introduction of EY, first FPIES reaction, and diagnosis were 208 days (IQR, 188–223.5), 236 days (IQR, 221.5–242.8), and 282 days (IQR, 252.8–307.5), respectively. There was no difference in baseline characteristics, including serum IgE specific to EY, egg white, and ovomucoid, between the included and excluded patients, except for age at diagnosis (Table 1).

**Figure 1:**
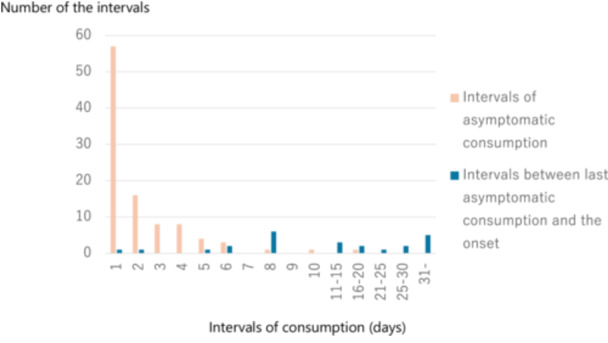
Comparison of ingestion intervals. Bars indicate the number of intervals during asymptomatic egg yolk consumption (orange, *n* = 99) and that between the last asymptomatic egg yolk consumption and onset (blue, *n* = 24).

Table 2 presents the days of EY consumption from its introduction to the onset of FPIES. The average interval during the asymptomatic consumption period was 2.2 ± 2.3 (median, 1; IQR, 1–3; 95% confidence interval [CI], 1.75–2.68) days. The average interval between the last asymptomatic consumption and onset of FPIES was 17.1 ± 12.7 (median, 13; IQR, 8–29; 95% CI, 11.70–22.46) days. The statistical power for this analysis was calculated as 1.00. Figure 1 summarizes the results. Thus, the interval between the last asymptomatic consumption and onset of FPIES was significantly longer than that during the asymptomatic period (*p* < 0.0001).

**Table 2:** Days of egg yolk consumption from the first introduction to the onset.

Case no.	Number of asymptomatic consumptions before onset	Days																																																
		1	2	3	4	5	6	7	8	9	10	11	12	13	14	15	16	17	18	19	20	21	22	23	24	25	26	27	28	29	30	31	32	33	34	35	36	37	38	39	40	41	42	43	….	51	52	53	54	55
1	7	○	○	○	○	○	○	○																																			•							
2	2	○	○																																			•												
3	7	○	○	○	○				○	○		○								•																														
4	5	○	○		○				○					○																										•										
5	8	○		○		○		○			○						○			○		○								•																				
6	6	○	○	○					○	○				○															•																					
7	6	○	○	○	○			○	○						•																																			
8	9	○	○	○	○	○	○	○	○	○																																							•	
9	2	○		○																																					•									
10	1	○					•																																											
11	3	○			○	○								•																																				
12	10	○		○		○		○		○		○		○		○		○		○																		•												
13	2	○	○	•																																														
14	5	○	○						○				○				○						•																											
15	4	○	○				○						○																														•							
16	6	○	○	○																		○	○										○														•			
17	7	○	○	○	○	○	○	○																								•																		
18	3	○				○					○															•																								
19	4	○	○	○								○								•																														
20	5	○				○			○					○			○								•																									
21	5	○	○	○	○	○																														•														
22	4	○	○			○			○		•																																							
23	5	○	○	○	○	○										•																																		
24	7	○	○	○	○	○	○	○									•																																	

*Note*: ○, asymptomatic egg yolk consumption; •, first episode of food protein‐induced enterocolitis syndrome caused by egg yolk.

## 4 DISCUSSION

We demonstrated for the first time that EY‐FPIES develops following prolonged cessation of regular EY consumption. Two‐thirds of patients were excluded due to incomplete data. However, there were mostly no differences in baseline characteristics between the included and excluded patients. The only difference was the age at diagnosis, possibly due to a recent tendency of early referral from primary pediatricians to our hospital.

To date, several authors have reported relapse of FPIES after confirming tolerance acquisition by a negative OFC. Argiz et al. reported that some patients with FPIES who were negative or inconclusive for the OFC experienced an acute FPIES reaction during the first subsequent culprit food exposure within a median of 8 days (range, 4–14 days) from the OFC.^4^ Moreover, Barni et al. reported that 4.5% of solid FPIES relapsed after an average of 3.5 ± 3.1 asymptomatic ingestions with a time latency of 30.1 ± 24.8 days from the negative OFC.^5^ Jacobs et al. suggested the importance of regular consumption of causative foods after a negative OFC.^6^ In contrast, in a study investigating the prevention of egg allergy in high‐risk infants with eczema (PETIT study), no patients vomited in a manner suggestive of FPIES during the daily intake of hen's egg powder.^7^ These reports support our finding that a prolonged interval of causative food ingestion is associated with the onset of FPIES.

Our findings suggest that regular and frequent EY intake following its initial introduction prevents EY‐FPIES in infants. Frequent administration of food proteins reduces the development of immediate‐type food allergies to peanuts and hen's egg possibly attributed to the induction of immunotolerance.^7^, ^8^ Additionally, the effectiveness of frequent low‐dose consumption of the causative foods has been reported in individuals who had already been diagnosed with FPIES.^9^ Histopathological studies have demonstrated the presence of activated T cells in the gastrointestinal lamina propria of patients with FPIES.^10^ Additionally, human leukocyte antigen‐DR expression is increased on dendritic cells in patients with FPIES.^11^ FPIES reactions are associated with a Th2‐skewed cytokine profile that is reflected in elevated serum levels of the thymus and activation‐regulated chemokines during acute FPIES reaction.^12^ Theses findings suggest a critical role of both T cells and antigen presenting cells in the development of FPIES. In animal models, a single high dose administration or consecutive small doses of antigen for 5–7 days induces oral tolerance.^13^ Frequent exposure to antigens induces peripheral regulatory T cells (pTreg) and anergy or exhaustion of effector T cells, which is critical for the establishment of immunotolerance to food allergens in model mice.^13^, ^14^, ^15^ However, pTreg possesses the plasticity and ability to convert to effector T cells, which plays a role in the development of food allergies and autoimmune diseases.^16^, ^17^ Thus, we speculate that a long interval of ingestion allows for the reprogramming of pTreg to effector T cells and restore the function of anergic or exhausted effector T cells. Additionally, as FPIES typically affects newborns and infants and enters remission after a long elimination time of one to several years, immaturity of the gastrointestinal tract may be a nonimmunological factor involved in the development of FPIES.^13^, ^18^

EY was introduced at a median age of 6 months and consumed a median of five times within a median of 25.5 days before FPIES symptoms developed (Table 1). The introduction of boiled EY at 5–6 months of age is currently recommended by the Japanese Guidelines for Food Allergy 2020 and Guidance of Nursing and Weaning (2019 version https://www.mhlw.go.jp/content/11908000/000496257.pdf in Japanese), although the 2007 version recommends the introduction of boiled EY at 7–8 months of age.^19^ EY is customarily introduced at a very small amount that is expressed as “almost equivalent to a cup of ear pick” and gradually increased to a whole EY in 1–2 months. Notably, there were no cases of rice‐associated FPIES in our hospital despite the introduction of rice gruel at 5–6 months of age. This is contrast with the data from Australia, where rice is the most prevalent cause of solid FPIES.^20^ We hypothesize that Japanese children may be less likely to develop rice‐induced FPIES due to the frequent exposure to rice as a daily consumed staple food. As EY is an easily available food item in the weaning diet, most guardians in Japan serve EY to their infants every 1–3 days. As the first FPIES symptoms develop before the age of 8 months, it is practical to serve EY every 1–3 days up to this age. Regular consumption of food proteins is also recommended for primary prevention of IgE‐mediated food allergy particularly in sensitized individuals, although optimal interval has not been elucidated.^21^, ^22^

One limitation of our study was that it was a retrospective review of a small number of patients from a single institution. Nevertheless, the present study had enough statistical power to derive our conclusion. We enrolled only patients with EY‐FPIES who possessed complete information regarding the date of EY consumption before the onset. Thus, we cannot completely exclude the possibility of selection bias. However, there were no differences in baseline characteristics between the included and excluded patients, suggesting that the present data can be generalized to most of our patients with EY‐FPIES. The optimal frequency and duration of EY consumption should be validated in large‐scale studies. Although a prospective study is ideal, it is difficult to conduct due to the rarity of the disease and the heavy burden on guardians.

## 5 CONCLUSION

The development of EY‐FPIES was associated with prolonged cessation of regular EY consumption. Consumption of EY at short intervals, for example, every 1–3 days, before 8 months of age may be important for preventing EY‐FPIES. Given the rarity of the disease, large‐scale prospective controlled studies are essential to determine optimal exposure frequency. These studies, however, will be challenging due to the potential guardian burden and will necessitate collaboration with obstetric facilities, regional pediatric centers and primary physicians, and community healthcare centers.

## CONFLICT OF INTEREST STATEMENT

The authors declare no conflicts of interest.
